# Comparison study on k-word statistical measures for protein: From sequence to 'sequence space'

**DOI:** 10.1186/1471-2105-9-394

**Published:** 2008-09-23

**Authors:** Qi Dai, Tianming Wang

**Affiliations:** 1 Department of Applied Mathematics, Dalian University of Technology, Dalian 116024, PR China

## Abstract

**Background:**

Many proposed statistical measures can efficiently compare protein sequence to further infer protein structure, function and evolutionary information. They share the same idea of using *k*-word frequencies of protein sequences. Given a protein sequence, the information on its related protein sequences hasn't been used for protein sequence comparison until now. This paper proposed a scheme to construct protein 'sequence space' which was associated with protein sequences related to the given protein, and the performances of statistical measures were compared when they explored the information on protein 'sequence space' or not. This paper also presented two statistical measures for protein: *gre.k *(generalized relative entropy) and *gsm.k *(gapped similarity measure).

**Results:**

We tested statistical measures based on protein 'sequence space' or not with three data sets. This not only offers the systematic and quantitative experimental assessment of these statistical measures, but also naturally complements the available comparison of statistical measures based on protein sequence. Moreover, we compared our statistical measures with alignment-based measures and the existing statistical measures. The experiments were grouped into two sets. The first one, performed via ROC (Receiver Operating Curve) analysis, aims at assessing the intrinsic ability of the statistical measures to discriminate and classify protein sequences. The second set of the experiments aims at assessing how well our measure does in phylogenetic analysis. Based on the experiments, several conclusions can be drawn and, from them, novel valuable guidelines for the use of protein 'sequence space' and statistical measures were obtained.

**Conclusion:**

Alignment-based measures have a clear advantage when the data is high redundant. The more efficient statistical measure is the novel *gsm.k *introduced by this article, the *cos.k *followed. When the data becomes less redundant, *gre.k *proposed by us achieves a better performance, but all the other measures perform poorly on classification tasks. Almost all the statistical measures achieve improvement by exploring the information on 'sequence space' as word's length increases, especially for less redundant data. The reasonable results of phylogenetic analysis confirm that *Gdis.k *based on 'sequence space' is a reliable measure for phylogenetic analysis. In summary, our quantitative analysis verifies that exploring the information on 'sequence space' is a promising way to improve the abilities of statistical measures for protein comparison.

## Background

Over the past few decades, major advances in the field of molecular biology, coupled with advances in genomic technologies, have led to an explosive growth of biological sequences databases. For example, there are several well-known databases about protein: Pfam [1] (a secondary database for multiple alignments and profile hidden Markov models), SCOP [2] (a secondary database containing protein family and structural information), Swiss-Prot [3] (primary database of protein sequences), and Protein Information Resource (PIR) [4] (primary database of protein sequences). This deluge of databases, in turn, produces new questions to analyze protein sequences such as how to classify protein sequences, induce their evolutionary information, and predict their structures.

Among protein sequence analysis, some important computational methods are similarity search, phylogenetic analysis and sequence classification. The similarity search [5-7] is to search a database of known function sequences and uses the structures and functions of the most closely matched known sequences to analyze the structure and function of query sequence. Phylogenetic analysis [8-12] is the study of the evolutionary history among species. It can also provide useful information for pharmaceutical researchers to determine which species share the medicinal qualities [13]. Classification protein [14,15] is to get a biologically meaningful partition. It has several advantages: when proteins are grouped into a family, it can provide us some clues about the general features of this family and evolutionary evidence of proteins, and further infer the biological function of a new sequence by its similarity to some function-known sequences. Moreover, protein classification can be used to facilitate protein three-dimensional structure discovery, which is very important for understanding proteins' functions. However, these computational methods heavily rely on the (dis)similarity measures defined among biological sequences.

Because of the importance of research into (dis)similarity measures, numerous efficient algorithms have been developed, but challenges remain. Moreover, we believe that further improvements in the (dis)similarity measures will allow us to design more effective tools, which can help us to look back more deeply in evolutionary time. One kind of the most common dissimilarity measures in this area is edit distance by aligning two sequences. It is defined as the required number of insertions, deletions, and replacements of characters from the first protein sequence to obtain the second protein sequence. But this measure is encountered with difficulties: (i) computation with regard to large biological databases [16,17]; (ii) the score schemes chosen [16]. Therefore, alignment-free measures are actively pursued to overcome the limitations of protein analysis by alignment.

Up to now, many efficient alignment-free measures for sequences comparison have been proposed, but they are still in the early development compared with alignment-based methods. One of the comprehensive reviews [16] reported several concepts of (dis)similarity measures, such as Euclidean distance [18], Mahalanobis distances [19], Kullback-Leibler discrepancy [20], Cosine distance [21] and Pearson's correlation coefficient [22]. Recently, several novel alignment-free measures have been designed for protein sequences analysis, such as S1 and S2 [23], W-metric [14], Universal Similarity Metric [15], Local decoding [24], CLUSS [25] and Long Short-Term Memory [26].

Among the statistical measures, each sequence is mapped into an *n*-dimensional vector according to its *k*-word frequencies. Linear Algebra theory is further employed to define the similarity score between sequences represented in vector spaces. The *kld *extended by Wu et al. (2001) is computed in terms of two vectors of relative frequencies of *k*-words over a sliding window from two given DNA sequences. However, in an application where some entries of vectors are equal to 0 or 1, *kld *becomes unsuitable. In this paper, we present two statistical measures to overcome the limitation of the measure *kld*. The contents can be summarized as follows:

1. We present a scheme to build protein 'sequence space' based on the score or amino acid substitution matrices and calculate *k*-word frequencies of protein 'sequence space'.

2. Two statistical measures *gre.k *and *gsm.k*, as the extended Jensen-Shannon Divergence, are proposed. They are based on *k*-word frequencies and Jensen-Shannon Divergence. Although these two concepts are not new, their generalizations result in the novel aspect of these measures. Particularly, the statistical measure, *Gdis.k*, is proved to be a valid distance measure.

3. Our measures are applied to extensive tests, e.g., protein sequence classification and phylogenetic analysis. The performances of our measures are compared with alignment-based measures and the existing statistical measures. Through the experiments, we want to address the following questions with the aid of well known statistical index: (A) how well our statistical measures perform compared with the existing statistical measures and alignment-based ones; (B) which statistical measure performs better when exploring the information on protein 'sequence space'; (C) whether the classification abilities of statistical measures depend on the choice of score matrices; (D) whether our measure, *Gdis.k*, is a valid distance measure for phylogenetic analysis.

## Results and discussion

### Classification of protein sequences

The proposed statistical measures are used to classify protein sequences. Several benchmark data sets of non-homologous protein structures have been developed in the last few years [27-30]. In this study, we have chosen the 36 protein domains of [27], the Rost and Sander data set (RS) and the 86 prototype protein domains of [28]. The Chew-Kedem data set (Additional file 1) was introduced in [27] and further studied in [31]. It consists of 36 protein domains drawn from PDB entries of three classes (alpha/beta, mainly-alpha, mainly-beta). Although this data set has been extensively used, the main draw back of this data is small size and high redundant. The Rost and Sander data set (RS126) (Additional file 2) was designed for the secondary structure prediction of proteins with a pair-wise sequence similarity of less than 25% [32], and it was used as a test data to evaluate the performances of similarity measures [33]. Here, we not only compare the proteins' secondary structures, but analyse the performance of (dis)similarity measures according to the proteins' classification as given by SCOP, release 1.69 [34]. We adopt this manually curated database as our gold standard containing expert knowledge for class level. This data set is trimmed to exclude sequences belonging to classes with <5 elements, thus a data set of 121 protein sequences, denoted by RS, is obtained. The Sierk-Pearson data set (Additional file 3), which consists of a non-redundant subset of 2771 protein families and 86 non-homologous protein families from the CATH protein domain database [35], was introduced in [28]. We estimate the homology of the data by employing CD-HIT program, which clusters protein databases at given sequence homology threshold [36]. Running CD-HIT with 70% homology threshold reveals that there are 29, 120, 86 sequences for data CK, RS and SP, respectively, below the homology threshold. This results clearly indicate that CK is high redundant, RS is low redundant, and SP is less redundant.

The experiments aim at evaluating the classification ability of the alignment-based measures and the statistical measures. The evaluation procedure is based on a binary classification of each protein pair, where 1 corresponds to the two protein sequences sharing the same class, 0 otherwise.

Given a data with size *n*, a *n *× *n *similarity/distance matrix can be obtained via each measure. The entries of the upper triangular similarity/distance matrix constitute a similarity vector of length $\left(\begin{array}{l} n\\ 2 \end{array}\right)$, which is used as prediction. Also, we can get a vector of length $\left(\begin{array}{l} n\\ 2 \end{array}\right)$ consisted of 1 and 0 as class labels. A perfect measure would completely separate negative from positive set. Of course, this does not happen in practice, and the classes are interspersed. The ROC curves permit to assess the level of accuracy of this separation without choosing any distance threshold for the separation point. In particular, the AUC will give us a unique number of the relative accuracy of each measure.

The measures evaluated are: alignment-based measures, our statistical measures (*gre.k *and *gsm.k*) and the six statistical measures outlined in Method section (*ed.k*, *cos.k*, *se.k*, *W.k*, *s1.k *and *s2.k*), where the alignment-based measures are Clustal X, Needleman-Wunsch (global alignment) or Smith-Waterman (local alignment) raw scores, with no correction for statistical significance, using ten score matrices (BLOSUM40, BLOSUM45, BLOSUM62, BLOSUM80, BLOSUM100, PAM40, PAM80, PAM120, PAM200, PAM250) and linear gap penalties or affine gap penalties, with a gap penalty of 8. All statistical measures based on *k*-word frequencies of protein sequence and protein 'sequence space' run with *k *from 1 to 4, where protein 'sequence space' is constructed based on the score matrix (BLOSUM40, BLOSUM45, BLOSUM62, BLOSUM80, BLOSUM100, PAM40, PAM80, PAM120, PAM200, PAM250). For each measure, separate tests are done with each combination of parameter values, and the best combination is chosen to represent the score in the performance. ROC curves are computed to evaluate and compare the performances of our methods and other (dis)similarity measures.

**Figure 1:**
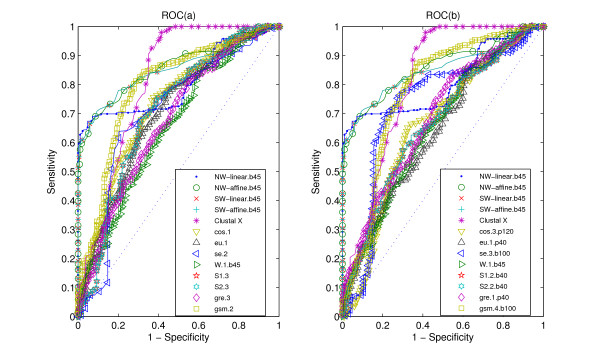
**ROC curves for data CK**. ROC (a) for our measures, alignment-based measures and other statistical measures, all the statistical measures are based on *k*-word frequencies of protein sequence, with the parameter values as suffix. ROC (b) for our measures, alignment-based measures and other statistical measures, all the statistical measures are based on *k*-word frequencies of protein 'sequence space', with the parameter values as suffix. All the abbreviations of (dis)similarity measures are illustrated in the "List of abbreviations" section. A random classifier would generate equal proportions of FP and TP classifications, which corresponds to the ROC diagonal (dashed line).

**Figure 2:**
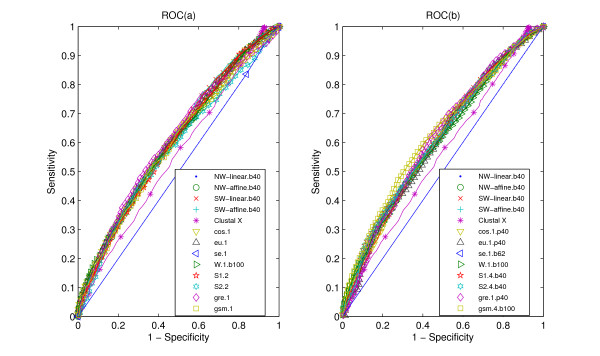
**ROC curves for data RS**. ROC (a) for our measures, alignment-based measures and other statistical measures, all the statistical measures are based on *k*-word frequencies of protein sequence, with the parameter values as suffix. ROC (b) for our measures, alignment-based measures and other statistical measures, all the statistical measures are based on *k*-word frequencies of protein 'sequence space', with the parameter values as suffix. All the abbreviations of (dis)similarity measures are illustrated in the "List of abbreviations" section. A random classifier would generate equal proportions of FP and TP classifications, which corresponds to the ROC diagonal (dashed line).

**Figure 3:**
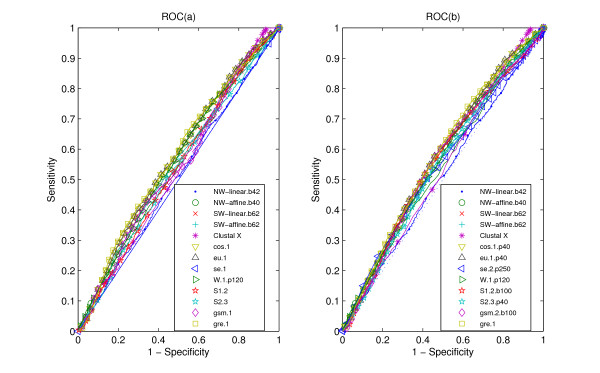
**ROC curves for data SP**. ROC (a) for our measures, alignment-based measures and other statistical measures, all the statistical measures are based on *k*-word frequencies of protein sequence, with the parameter values as suffix. ROC (b) for our measures, alignment-based measures and other statistical measures, all the statistical measures are based on *k*-word frequencies of protein 'sequence space', with the parameter values as suffix. All the abbreviations of (dis)similarity measures are illustrated in the "List of abbreviations" section. A random classifier would generate equal proportions of FP and TP classifications, which corresponds to the ROC diagonal (dashed line).

The ROC curves obtained for the classifications are presented in Figures 1, 2, 3. Figure 1(a), Figure 2(a) and Figure 3(a) denote the ROC curves of alignment-based measures and the statistical measures based on *k*-word frequencies of protein sequences. Figure 1(b), Figure 2(b) and Figure 3(b) denote the ROC curves of alignment-based measures and the statistical measures based on *k*-word frequencies of protein 'sequence space'. The better (dis)similarity measures have plots with higher values of sensitivity for equal values of specificity, resulting in higher values for the areas under the curves. The AUC value is typically used as a measure of overall discrimination accuracy. Table 1 provides the areas under ROC curves (AUC) obtained from all the (dis)similarity measures for data sets CK, RS and SP.

**Table 1:** The entries of AUC for Data CK, RS and SP

**CK**		**RS**		**SP**	
**Method**	**Area**	**Method**	**Area**	**Method**	**Area**
NW-linear.b45	0.808	NW-linear.b40	0.605	NW-linear.b62	0.509
NW-affine.b45	**0.860**	NW-affine.b40	**0.614**	NW-affine.b40	0.540
SW-linear.b45	0.850	SW-linear.b40	0.600	SW-linear.b62	**0.548**
SW-affine.b45	0.850	SW-affine.b40	0.600	SW-affine.b62	**0.548**
Clustal X	0.807	Clustal X	0.555	Clustal X	0.535

***k-word FPS^a^***		***k-word FPS^a^***		***k-word FPS^a^***	

**Method**	**Area**	**Method**	**Area**	**Method**	**Area**

cos.1	0.729	cos.1	**0.609**	cos.1	0.569
eu.1	0.700	eu.1	0.607	eu.1	0.570
se.2	0.701	se.1	0.500	se.1	0.495
W.1.b45	0.652	W.1.b100	0.601	W.1.p120	0.559
s1.3	0.708	s1.2	0.581	s1.2	0.535
s2.3	0.708	s2.2	0.578	s2.3	0.530
gre.3	0.673	gre.1	0.607	gre.1	**0.572**
gsm.2	**0.791**	gsm.1	0.594	gsm.1	0.524

***k-word FPSS^b^***		***k-word FPSS^b^***		***k-word FPSS^b^***	

**Method**	**Area**	**Method**	**Area**	**Method**	**Area**

cos.3.p120	0.655	cos.1.p40	0.604	cos.1.p40	0.571
eu.1.p40	0.640	Eu.4.p80	0.603	eu.1.p40	0.570
se.3.b100	0.726	se.1.p250	0.501	se.2.p250	0.545
W.1.b45	0.652	W.1.b100	0.601	W.1.b100	0.559
s1.2.b40	0.667	s1.4.b40	0.607	s1.2.b100	0.554
s2.2.b40	0.667	s2.4.b40	0.607	s2.3.p40	0.545
gre.1.p40	0.683	Gre.4.b100	0.615	gre.1.p40	**0.575**
gsm.4.b100	**0.776**	gsm.3.b40	**0.627**	gsm.2.b100	0.557

The comparison of the areas under ROC curves (AUC) obtained from all the (dis)similarity measures for data CK, RS and SP. *k-word FPS*^*a *^denotes the *k*-word frequencies of protein sequences. *k-word FPSS*^*b *^denotes the *k*-word frequencies of protein 'sequence space'. All the abbreviations of (dis)similarity measures are illustrated in the "List of abbreviations" section.

#### Question A

In the CK experiment, Figure 1 and Table 1 show that alignment-based measures perform better than alignment-free measures. NW-affine.b45 outperforms other alignment-based measures, its area under ROC curve is 0.860. Among the statistical measures based on *k*-word frequencies of protein sequences, *gsm.2 *is clearly more efficient than other measures. Its area under ROC curve is 0.791. The next best measure is the *cos.1*, with the area under ROC curve 0.729, and the other measures lag behind. For the statistical measures based on *k*-word frequencies of protein 'sequence space', *gsm.4.b100 *is significantly better than other statistical measures, the *se.3.b100 *followed.

In the RS experiment, Figure 2 and Table 1 indicate that some statistical measures perform as well as alignment-based measures. By exploring the information on protein 'sequence space', the statistical measure, *gsm.k*, performs better than alignment-base measures. For the alignment-based measures, *NW-affine.b40 *performs better than other measures. As for the statistical measures based on *k*-word frequencies of protein sequences, *cos.1 *outperforms the other measures. Among the statistical measures based on *k*-word frequencies of protein 'sequence space', *gsm.3.b40 *is significantly better than all other measures, its area under ROC curve is 0.627, and the next best measure is *gre.4.b100*.

In the SP experiment, Figure 3 and Table 1 illustrate that some statistical measures defined by *k*-word frequencies of protein sequences outperform alignment-based measures. When the information on protein 'sequence space' is added, all the statistical measures, except for *se.k *and *s2.k*, perform better than alignment-base measures. For the alignment-based measures, *SW *measures perform better than *NW *measures. As for the statistical measures based on *k*-word frequencies of protein sequences, *gre.1 *outperforms other measures, which is followed by cos.1 and *eu.1*. Among the statistical measures based on *k*-word frequencies of protein 'sequence space', the area under ROC curve of *gre.1.p40 *is 0.575, better than other statistical measures, and the next best measures are the *cos.1.p40 *and *eu.1.p40*.

From the above three experiments, we can see that alignment-based measures have a clear advantage when the data is high redundant. The most efficient statistical measure is the novel *gsm.k *introduced by this report. When the data becomes less redundant, *gre.k *proposed by us achieves a better performance, but all the alignment-based and the existing measures perform poorly on all classification tasks. The inspection of the ROC curves themselves (Figures 1, 2, 3) further illustrates these comparisons between (dis)similarity measures.

#### Question B

The main goal of construction of protein 'sequence space' is to improve the classification ability of (dis)similarity measures by extracting the information on related protein sequences. However, it should be noted that not all the (dis)similarity measures are suitable for this scheme. In order to find which statistical measure is suitable for this scheme, we define a function *DAUC (measure, score matrix, k) *to evaluate whether the classification ability of (dis)similarity measures improve or not,

(1) $$\begin{array}{c} DAUC\;(measure,score\;matrix,k)=AUC\;(measure,score\;matrix,k)\\ -AUC\;(measure,k), \end{array}$$

where *AUC (measure, score matrix, k) *denotes the area under ROC curve of the statistical measure based on the *k*-word frequencies of protein 'sequence space', which is constructed based on the score matrix; *AUC (measure, k) *denotes the area under ROC curve of measure defined by the *k*-word frequencies of protein sequence.

**Figure 4:**
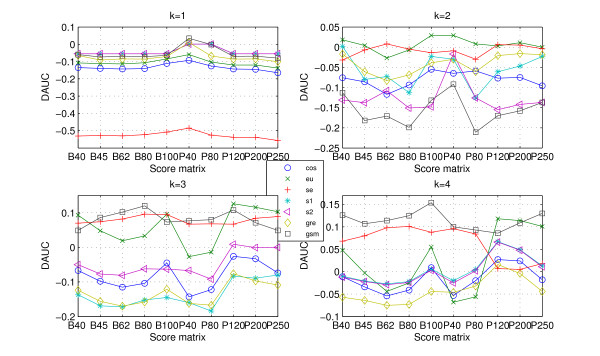
***DAUC *values for data CK**. The *DAUC *values of seven statistical measures for data CK. All statistical measures based on *k*-word frequencies of protein 'sequence space' run with *k *from 1 to 4, where protein 'sequence space' is constructed according to ten score matrices. One graph presents each word length (from 1 to 4).

**Figure 5:**
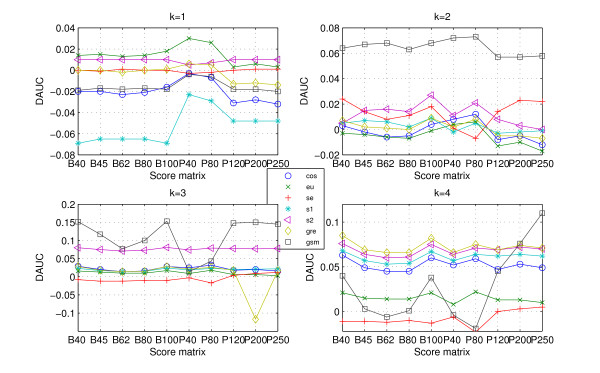
**DAUC values for data RS**. The *DAUC *values of seven statistical measures for data RS. All statistical measures based on *k*-word frequencies of protein 'sequence space' run with *k *from 1 to 4, where protein 'sequence space' is constructed according to ten score matrices. One graph presents each word length (from 1 to 4).

**Figure 6:**
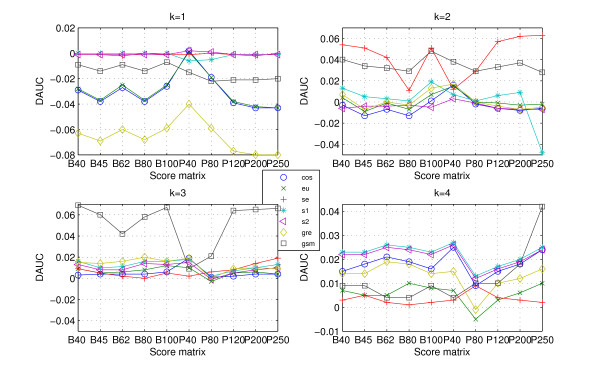
**DAUC values for data SP**. The *DAUC *values of seven statistical measures for data SP. All statistical measures based on *k*-word frequencies of protein 'sequence space' run with *k *from 1 to 4, where protein 'sequence space' is constructed according to ten score matrices. One graph presents each word length (from 1 to 4).

Judging from definition of *DAUC*, it is easier to recognize that if DAUC ≥ 0, utilizing protein 'sequence space' improves the classification ability of the (dis)similarity measures. The *DAUC *values for the data CK, RS and SP are presented in Figures 4, 5, 6.

As would be expected, the *DAUC *values of the different measures (Figures 4, 5, 6) show two clear trends: (i) the *DAUC *values increase from *k *= 1 to *k *= 4 for all three data sets. When the length of word is equal to 4, almost all the statistical measures' classification abilities are improved. It should be noted that the classification discrimination of statistical measures based on higher order word frequencies, such as *eu.k*, *se.k *and *cos.k*, worsens [14], because the high dimension of the frequency vectors and the relative low dimension of the sequences length itself cause the frequency vector *F *to be very sparse. Interestingly, the construction of protein 'sequences space' maintains the accuracy and overcomes the difficulty arising from higher order word; (ii) it is interesting to note that there is a dependency between usefulness of protein 'sequence space' and the level of data's redundant. When the data is high redundant such as CK, the 'sequence space' is more similar. Consequently, the (dis)similarity measures based on 'sequence space' achieve a little improvement (Figure 4 (k = 4)). But the accuracy of classification is also improved with word's length increasing. As for the less redundant data such as RS and SP, all the statistical measures based on 'sequence space' achieve significantly improvement when word's length increases to 4 (Figures 4, 5 (k = 4)).

#### Question C

**Figure 7:**
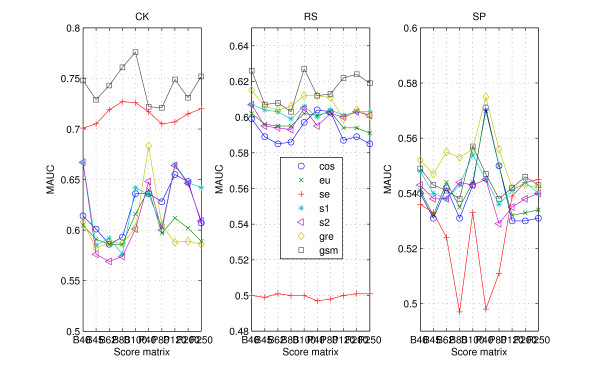
**MAUC values for data sets CK, RS and SP**. The *MAUC *values for the data CK, RS and SP, one for each data. All the statistical measures are based on *k*-word frequencies of protein 'sequence space', with ten score matrices to build protein 'sequence space'.

Using protein 'sequence space' contributes to the accuracy of protein classification. However, the construction of protein 'sequence space' relies heavily on the score matrix. In order to evaluate the influence of different score matrices, the function *MAUC(measure, score matrix) *is defined by

(2) $$\begin{array}{l} \text{MAUC(measure, score matrix)}\\ =\underset{1\leq \text{k}\leq \text{4}}{\text{max}}\left(\text{AUC(measure, score matrix,k)}\right) \end{array},$$

where *AUC (measure, score matrix, k) *denotes the area under ROC curve of the statistical measure based on the *k*-word frequencies of protein 'sequence space' that is built based on the score matrix. The *MAUC *values of all the statistical measures based on ten score matrices for three data sets are presented in Figure 7. Figure 7 largely confirms that the measures possess different performances based on different score matrices. The changes of *DAUC *for the data CK, RS and SP are similar. For BLOSUM score matrix, BLOSUM40 and BLOSUM100 perform better in improvement of the statistical measures' classification abilities. As for PAM score matrix, PAM120 or PAM250 improves the classification ability of all the (dis)similarity measures on the high redundant data more obviously, except for the measures *eu.k *and *gre.k*. PAM40 or PAM80 contributes to improve the classification ability of the (dis)similarity measures more obviously on the less redundant data.

### Phylogenetic analysis

Since *Gdis.k *is a statistical distance measure, it is further tested to analyze phylogenetic relationships. Given a set of protein sequences, their phylogenetic relationships can be obtained through the following main operations: firstly, the *k*-word frequencies of protein 'sequence space' are calculated; secondly, the statistical distances are calculated and arranged into a distance matrix; finally, the phylogenetic relationships is obtained by neighbor-joining program in the PHYLIP package [37].

**Figure 8:**
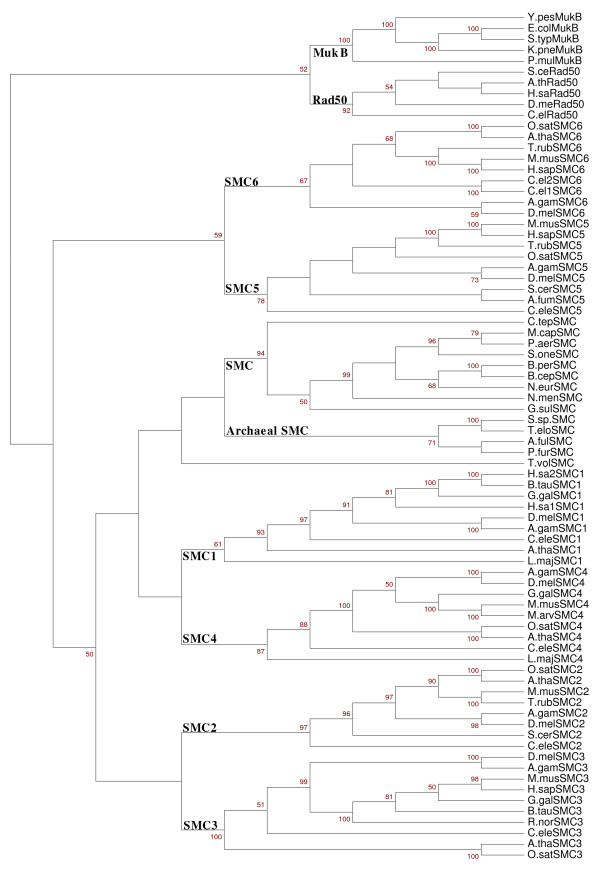
**The diagram of phylogenetic relationships**. Phylogenetic relationships are obtained by neighbor-joining program based on our statistical distance measure *Gdis.k *using all six SMC subfamilies, as well as the related MukB and Rad50. Bootstraps are based on 100 replications, and bootstrap values, lower than 50, are hidden.

A data set includes 68 SMC proteins, 5 Rad50 proteins and 5 MukB proteins (Additional file 4), which have been widely studied [38-42]. Our distance measure is applied to this data, and the results are shown in Figure 8. To assess the robustness of an estimated tree under perturbations of the input alignment, it is customary to perform a bootstrap analysis, where entire columns of the alignment are resampled with replacement. The bootstrap technique is employed to evaluate the tree topologies by resampling the sequence 100 times. We obtain the phylogenetic relationships drawn by MEGA program [11], bootstrap values, lower than 50, are hidden. Generally, an independent method can be developed to evaluate the accuracy of phylogenetic relationships, or the validity of phylogenetic relationships can be tested by comparing it with authoritative ones. Here, we adopt the latter one to test the validity of our measure.

#### Question D

Our results are quite consistent with the accepted taxonomy and authoritative ones [40-42] in the following three aspects. First of all, all the organisms are clearly separated from each other. Among the SMC proteins, it is consistently observed that SMC1 and SMC4 are grouped closely (there are the larger SMC subunits of the cohesin and condensin SMC heterodimers, respectively), and the smaller subunits, SMC3 and SMC2, appear to group closely. SMC5 and SMC6 are grouped together, which is consonant with that they heterodimerize as part of a DNA repair complex [42,43]. Secondly, it is obvious from this tree that the closest relatives to the SMC proteins are the Rad50 proteins, followed by MukB proteins. Many of these Rad50 superfamily proteins have the conserved N-terminal FKS (or FRS) motif (located before the Walker A site), which is presented in most of the SMC proteins [41]. Finally, among the SMC proteins, it is observed that SMC1 protein and SMC4 protein are closer to SMC proteins, followed by SMC2, SMC3, SMC5 and SMC6 [41,42]. It suggests that the duplication events giving rise to each subfamily must have occurred either before or very soon after the origin of eukaryotes. Since the rate of accepted amino acid substitution varies among different eukaryotic taxa within each subfamily. Condensin SMCs appear to show a higher substitution than cohesin SMCs, the mean distances within subfamilies of these proteins (averaged across all condensin and cohesin SMCs for each pairwise comparison between different organisms) are about half (0.54 ± 0.134) the corresponding distances between SMC5 and SMC6 proteins [41]. These reasonable results confirm that *Gdis.k *is a reliable distance measure for phylogenetic analysis.

## Conclusion

Prior to this research, the statistical measures are perceived as adequate for analysis of biological data mainly because of their flexibility and scalability with data set size. In particular, some of them are quantitatively compared for the recognition of SCOP relationships [14]. This article presents a novel way to compare protein sequences by exploring the information on 'sequence space' and two new statistical measures: *gre.k *and *gsm.k*. It offers the first systematic and quantitative experimental assessment of statistical measures based on protein sequence and protein 'sequence space', which naturally complements the many available comparisons based on protein sequences.

The accuracy of each (dis)similarity measure to classify protein sequence is assessed through the experiments on high redundant and less redundant data sets. The comparative index AUC is a good measure of overall accuracy of a classification scheme. The proposed statistical distance measure, *Gdis.k*, is further tested to analyze phylogenetic relationships.

As for the high redundant data, alignment-based measures have a clear advantage. *gsm.k*, followed by *cos.k*, is clearly more efficient among the existing statistical measures (Figure 1 and Table 1). When the data becomes less redundant, all the statistical measures, except for *se.k *and *s2.k*, outperform the alignment-based measures by exploring the information on protein 'sequence space', and *gre.k *proposed by us achieves the best performance (Figure 3 and Table 1). The scheme for constructing 'sequence space' can provide more information than the protein sequence only and contributes to the accuracy of protein classification, especially for the less redundant data sets such as RS and SP. Almost all the statistical measures based on 'sequence space' achieve significantly improvement when word's length increases to 4 (Figures 4, 5, 6). In addition, the reasonable results of phylogenetic analysis illustrate the validity of our distance measure for phylogenetic analysis.

Overall our comparison study highlights the necessity for alignment-free measures to extract more information as possible. Thus, this understanding can then be used to guide development of more powerful measures for protein sequence comparison with future possible improvement on evolutionary, structure and function study. But, it is worthy to note that although exploring the information on 'sequence space' improves the classification ability of some (dis)similarity measures, they all perform very poorly, near random classification values of 0.5 for less redundant data. That is to say, they may be useless in practice. So we expect a further investigation on the statistical methods, especially for low redundant datasets

## Methods

### Word statistics

#### Word statistics in protein sequence

There is a large body of literatures on word statistics [45], where sequences are interpreted as a succession of symbols and are further analyzed by representing the frequencies of its small segments. A *k*-word is a series of *k *consecutive letters in a sequence. The *k*-word statistical analysis consists of counting occurrences of *k*-words in a given sequence. For a sequence *s*, the count of a k-word *w*, denoted by *c(w)*, is the number of occurrence of *w *in the sequence *s*. The standard approach for counting *k*-words in a sequence of length *m *is to use a sliding window of length *k*, shifting the frame one base at a time from position 1 to *m-k+1*. In this method, *k*-words are allowed to overlap in the sequence. In this way, a sequence can be represented by an *n*-dimensional vector $C_{k}^{s}$ made up of *k*-word counts

(3) $$C_{k}^{s}=\left(c(w_{k,1}),c(w_{k,1}),\cdots ,c(w_{k,n})\right),$$

where *n *is the number of all possible *k*-words. For example, consider the protein sequence *s *= *VCST*, we can obtain the vector made up of *2*-word counts

(4) $$C_{2}^{s}=\left(c(VC),c(CS),c(ST)\right)=(1,1,1).$$

The frequencies of *k*-words, $F_{k}^{s}$, can he calculated by

(5) $$\begin{array}{c} F_{k}^{s}=\left(f(w_{k,1}),f(w_{k,1}),\cdots ,f(w_{k,n})\right)\\ =\left(\frac{c(w_{k,1})}{m-k+1},\frac{c(w_{k,2})}{m-k+1},\cdots ,\frac{c(w_{k,n})}{m-k+1}\right) \end{array}.$$

#### Word statistics in protein 'sequence space'

The number of possible protein sequences is enormous. When a protein sequence is given, we are interested in its related proteins, and we denote them as the 'sequence space' of the given protein.

Substitution matrices represent similarity of amino acids, where each entry *m*_*ij *_of a substitution matrix [*m*_*ij*_] represents the 'normalized probability' (score) that amino acid *i *can mutate into amino acid *j*. Let *i *ℵ *j *denotes that the amino acids *i *and *j *are similar. Usually, two amino acids *i *and *j *are considered similar if *m*_*ij *_> 0. That is to say

(6) *i *ℵ *j *if *m*_*ij *_> 0 ∀ *i*, *j *∈ Ω

where Ω = {A,C,D,E,F,G,H,I,K,L,M,N,P,Q,R,S,T,V,W,Y}. Note that the substitution matrices are symmetric matrices, i.e., *a *being similar to *b *implies that *b *is similar to *a*. But this similarity of amino acids is not a transitive relation. For example, *a *is similar to *b *and *b *is similar to *c*, but *a *is not similar to *c*. Therefore, 20 amino acids are not possibly classified into several similarity classes according to this property.

**Figure 9:**
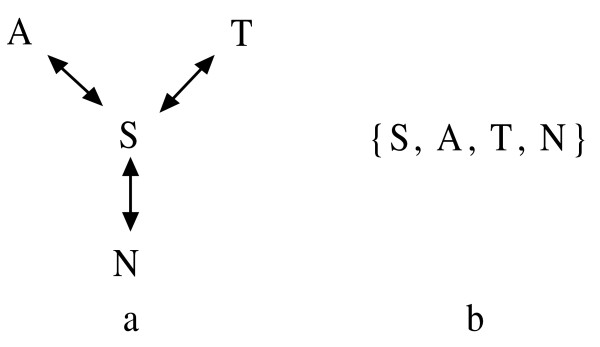
**Representation of a star set**. a: the diagram of star set, *S *is similar to *A*, *T *and *N *in BLOSUM62 substitution matrix, and *S *is the midpoint; b: the star set consists of the midpoint *S *and vertices *A*, *T *and *N*.

We shall bypass the above similarity classes and consider a new star set which is easily to implement. A star set assumes that the properties are known between vertices and center. We can construct a star set including all the vertices and the center, and specifically write the center as the first element of the set to distinguish one set from the others. For example, *S *is similar to *A*, *T *and *N *in BLOSUM62 substitution matrix, so *S *is the center and they can constitute a star set {*S*, *A*, *T*, *N*} presented in Figure 9. For writing convenience, we write the star set {*S*, *A*, *T*, *N*} as ℵ*S *= {*x *| *x *ℵ *S*, *x *∈ Ω}. With the aid of star set, 20 amino acids can be partitioned into 20 star sets presented in Table 2 based on BLOSUM62 substitution matrix.

**Table 2:** The 20 star sets

Matrix	Star set				
BLOSUM62	{AS}	{C}	{DNE}	{EDQK}	{FYW}
	{G}	{HNY}	{IMLV}	{KEQR}	{LMIV}
	{MILV}	{NSDH}	{P}	{QERK}	{RQK}
	{SATN}	{T S}	{VMIL}	{WFY}	{YHFW}

With help of the star set, 20 amino acids are partitioned into 20 star sets based on substitution matrix BLOSUM62.

Our work derives a way to build 'sequence space' with the help of star set. From the definition of star set, we know that each amino acid corresponds a star set. For example, the star set of the amino acid *S *is ℵ*S *= {*S*, *A*, *T*, *N*} according to BLOSUM62 substitution matrix. Given two protein sequences *P *= *p*_1_*p*_2 _⋯ *p*_*n *_and *Q *= *q*_1_*q*_2 _⋯ *q*_*n*_,

(7) ∀ *p*_*i *_∈ *P*, *q*_*i *_∈ *Q*, if *p*_*i *_∈ ℵ*q*_*i *_⇒ *P *ℑ *Q*

where *P *ℑ *Q *denotes that the protein sequences *P *and *Q *are related. Given a protein sequence *s*, its 'sequence space', denoted by *SP*_*s*_, is defined as follows:

(8) *SP*_*s *_= {*P *| *P *ℑ *s*, *length*(*P*) = *length*(*s*)}

where *P *is a protein sequence, *length(P) *denotes the length of the protein sequence *P*. The protein 'sequence space' can be constructed as follows: for each protein sequence, beginning with the first amino acid, we scan through the protein sequence and substitute the star sets for amino acids at each position, respectively. Thus a special set of protein sequences is obtained, which is denoted as the 'sequence space' of the protein sequence. For example, given a protein sequence *s *= *VCST*, the star sets of *V*, *C*, *S*, and *T *are {*V*, *M*, *I*, *L*}, {*C*}, {*S*, *A*, *T*, *N*} and {*S*, *A*, *T*, *N*} according to BLOSUM62 substitution matrix, and the 'sequence space' of protein *s *is {*V*, *M*, *I*, *L*}-{*C*}-{*S*, *A*, *T*, *N*}-{*T*, *S*}.

Once the protein 'sequence space' is built, the *k*-word frequencies of 'sequence space' can be computed similarly. A segment of *k *symbols from a finite alphabet, ***A ***with 20 letters, is designated a *k*-word. The set *W*_*k *_= (*w*_*k*,1_, *w*_*k*,2_, ⋯, *w*_*k*, *Y*_) consists of all possible *k*-words that can be extracted from protein 'sequence space', and has Y elements, where *Y *= 20^*k*^. The count of *k*-words in protein 'sequence space', denoted by $C_{k}^{sp_{s}}=\left(c^{sp_{s}}(w_{k,1}),c^{sp_{s}}(w_{k,1}),\cdots ,c^{sp_{s}}(w_{k,Y})\right)$ can be calculated by taking a sliding window with *k*-wide and scanning through the protein 'sequence space'. For example, considering the protein sequence *s *= *VCST*, its 'sequence space' is {*V*, *M*, *I*, *L*}-{*C*}-{*S*, *A*, *T*, *N*}-{*T*, *S*}, we can get a vector of *2*-word counts

(9) $$\begin{array}{c} C_{2}^{SP_{s}}=\left(c^{SP_{s}}(VC),c^{SP_{s}}(MC),\cdots ,c^{SP_{s}}(NS)\right)\\ =(1,1,\cdots ,1) \end{array}$$

Similarly, one can then calculate *k*-word frequencies of protein 'sequence space', denoted as $F_{k}^{sp_{s}}$, by

(10) $$\begin{array}{c} F_{k}^{SP_{s}}=\left(f^{SP_{s}}(w_{k,1}),f^{SP_{s}}(w_{k,1}),\cdots ,f^{SP_{s}}(w_{k,n})\right)\\ =\left(\frac{c^{SP_{s}}(w_{k,1})}{\sum_{t=1}^{Y}c^{SP_{s}}(w_{k,t})},\frac{c^{SP_{s}}(w_{k,2})}{\sum_{t=1}^{Y}c^{SP_{s}}(w_{k,t})},\cdots ,\frac{c^{SP_{s}}(w_{k,Y})}{\sum_{t=1}^{Y}c^{SP_{s}}(w_{k,t})}\right). \end{array}$$

### Statistical distance measures

#### Previous (dis)similarity measures

We first describe the six previous statistical measures for biological sequences.

Many statistical measures for sequence comparison are to fix a short word length *k*, compute the frequencies of all *k*-words in each sequence, and assess the similarity of the two frequency vectors.

##### 1. Euclidian distance (*ed.k*)

The Euclidian distance is one of the most common dissimilarity measures of biological sequences. The dissimilarity score between two protein sequences *X *and *Y *is the Euclidian distance between their *k*-word frequencies $F_{k}^{A}=\left(f(w_{k,1}^{A}),f(w_{k,1}^{A}),\cdots ,f(w_{k,n}^{A})\right)$ and $F_{k}^{B}=\left(f(w_{k,1}^{B}),f(w_{k,1}^{B}),\cdots ,f(w_{k,n}^{B})\right)$[18]

(11) $$\begin{array}{c} ed.k(X,Y)={\left(F_{k}^{X}-F_{k}^{Y}\right)}^{'}\cdot (F_{k}^{X}-F_{k}^{Y})\\ =\sum_{t=1}^{n}{\left(f(w_{k,t}^{X})-f(w_{k,t}^{Y})\right)}^{2} \end{array}.$$

##### 2. Cosine of the angle (*cos.k*)

In order to derive estimation of relatedness from the vector definitions of biological sequences, Stuart et al. (2002) proposed the pair-wise cosine for generating accurate gene and species phylogenies from whole genome sequences.

(12) $$\begin{array}{l} \cos.k(X,Y)=-\ln[(1+\cos(X,Y))/2],\\ \cos(X,Y)=\frac{\sum_{t=1}^{n}f(w_{k,t}^{X})\cdot f(w_{k,t}^{Y})}{\sqrt{\sum_{t=1}^{n}{\left(f(w_{k,t}^{X})\right)}^{2}}\cdot \sqrt{\sum_{t=1}^{n}{\left(f(w_{k,t}^{Y})\right)}^{2}}}. \end{array}$$

Cosine is a standard measure of vector similarity, and its application for this purpose can be understood intuitively.

##### 3. Standardized Euclidean distance (*se.k*)

The above measures explore the use of Euclidean distances and correlations between k-word frequencies representations of sequences. Standardized Euclidean distance takes into account the data covariance structure

(13) $$\begin{array}{c} se.k(X,Y)={\left(F_{k}^{X}-F_{k}^{Y}\right)}^{'}\cdot {\left[diag(s_{11},\cdots s_{nn})\right]}^{-1}(F_{k}^{X}-F_{k}^{Y})\\ =\sum_{t=1}^{n}\frac{f(w_{k,t}^{X})-f(w_{k,t}^{Y})}{s_{tt}} \end{array},$$

where S = [s_*ij*_] represents the covariance matrix of *k*-word frequencies. The standard Euclidean distance forces cov (*f*_*i*_, *f*_*j*_) = 0 for *i *≠ *j*. Therefore, in this distance measure the correlations between different *k*-words are ignored and only the same *k*-word variances are accounted for. The standard Euclidean distance was first proposed for sequence comparison by Wu et al. (1997).

##### 4. Kullback-Leibler discrepancy (*kld*)

Let *P*_1 _and *P*_2 _be two probability frequencies on a universe *X*, the Kullback-Leibler divergence (*kld*) or the relative entropy, denoted as *kld(P1, P2)*, of *P*_1 _with respect to *P*_2 _is defined by the Lebesgue integral [46],

(14) $$kld(P_{1},P_{2})=\int_{X}\log(\frac{d(P_{1})}{d(P_{2})})d(P_{1}).$$

Although relative entropy is not a true metric, it satisfies many important mathematical properties. Wu et al. (2001) have applied Kullback-Leibler discrepancy to compare DNA sequences based on the frequencies of all *k*-words.

##### 5. W-metric (*W.k*)

In an application where the covariance matrices *S *chosen in standard Euclidean distance is replaced by amino acid substitution matrices, Vinga et al. (2004) proposed and demonstrated the use of W-metric as a novel k-word composition metric

(15) $$\begin{array}{l} W.k(X,Y)={\left(F_{k}^{X}-F_{k}^{Y}\right)}^{'}\cdot W\cdot (F_{k}^{X}-F_{k}^{Y})\\ =\sum_{i=1}^{n}\sum_{j=1}^{n}(f(w_{k,i}^{X})-f(w_{k,i}^{Y}))\cdot (f(w_{k,j}^{X})-f(w_{k,j}^{Y}))\cdot w_{ij} \end{array}$$

where *W *is amino acid substitution matrices such as BLOSUM and PAM. *W.k *is a distance defined between protein sequences, which bridges between alignment-based metrics and measures based solely on k-word composition.

##### 6. *S*_1 _and *S*_2 _(*s1.k *and *s2.k*)

*S*_1_and *S*_2 _are statistical measures for protein sequences based on the concept of comparing the similarity between the *k*-word appearances [23]. If the set $W_{k}^{X}=\left(w_{k,1}^{X},w_{k,2}^{X},\cdots ,w_{k,n}^{X}\right)$ and $W_{k}^{Y}=\left(w_{k,1}^{Y},w_{k,2}^{Y},\cdots ,w_{k,n}^{Y}\right)$ consist of all possible *k*-words that can be extracted from proteins *X *and *Y*, respectively, *S*_1 _and *S*_2 _can be computed by

(16) s1.k=c×|Match(WkX,WkY)|(|Word(WkX)|+|Word(WkY)|)s2.k=c×|Match(WkX,WkY)|(|Word(WkX)|+|Word(WkY)|−|length(X)−length(Y)|)

where |*Match*(${\text{W}}_{\text{k}}^{\text{X}}$, ${\text{W}}_{\text{k}}^{\text{Y}}$)| is the total number of *k*-words shared by two proteins *X *and *Y*, constant c is a normalizing factor; |*Word*(${\text{W}}_{\text{k}}^{\text{X}}$)| and |*Word*(${\text{W}}_{\text{k}}^{\text{Y}}$)| denote the total numbers of occurred *k*-words in proteins *X *and *Y*.

#### Novel statistical distance measures

We describe two novel statistical measures for protein sequences comparison based on *k*-word frequencies.

##### 1. Generalized relative entropy (*gre.k*)

Relative entropy is the most important concept in both statistical biology and information theory. It has been explored as similarity measures such as *kld *and *SimMM *[17,20] to compare biological sequences. However, in an application where *P*_*k *_is equal to 0 or 1, *kld*(*P*^1^, *P*^2^) → ∞. So the similarity measure *kld *becomes unsuitable. For such an application, we generalize relative entropy with the help of Jensen-Shannon Divergence, denoted by *gre.k*, by

(17) $$gre.k(X,Y)=\sum_{t=1}^{n}f(w_{k,t}^{X})\cdot \log_{2}\left(\frac{2\cdot f(w_{k,t}^{X})}{f(w_{k,t}^{X})+f(w_{k,t}^{Y})}\right).$$

Now, if $f(w_{k,t}^{X})$ is equal to 0 and 1,

(18) $$f(w_{k,t}^{X})\cdot \log_{2}\left(\frac{2\cdot f(w_{k,t}^{X})}{f(w_{k,t}^{X})+f(w_{k,t}^{Y})}\right)=0.$$

So *gre.k *can deal with all kinds of *k*-word frequencies.

##### 2. Gapped similarity measure (*gsm.k*)

From the definition of *gre.k*, it is worthy to note that the frequencies of *k*-words that are present in both sequences have different impact on the *gre.k*. But the frequencies of *k*-words that are present in only one sequence have no contribution to *gre.k*. Because if $f(w_{k,t}^{X})$ or $f(w_{k,t}^{Y})$ is equal to 0,

$$\begin{array}{ccc} f(w_{k,t}^{X})\cdot \log_{2}\left(\frac{2\cdot f(w_{k,t}^{X})}{f(w_{k,t}^{X})+f(w_{k,t}^{Y})}\right)=0 & \text{or} & f(w_{k,t}^{Y})\cdot \log_{2}\left(\frac{2\cdot f(w_{k,t}^{Y})}{f(w_{k,t}^{X})+f(w_{k,t}^{Y})}\right)=0 \end{array}.$$

Similarly, the measures *S*_1 _and *S*_2 _focus on the appearances of k-words but ignore their frequencies. Motivated by extracting the information from all the *k*-words, we investigate a novel statistical measure for protein sequence comparison, called the gapped similarity measure

(19) $$\begin{array}{l} gsm.k(X,Y)=\sum_{t=1}^{n}score(f(w_{k,t}^{X}),f(w_{k,t}^{Y}))\\ score(f(w_{k,t}^{X}),f(w_{k,t}^{Y}))\\ =\{\begin{array}{cc} f(w_{k,t}^{X})\cdot \log_{2}\left(\frac{2\cdot f(w_{k,t}^{X})}{f(w_{k,t}^{X})+f(w_{k,t}^{Y})}\right) & if\;f(w_{k,t}^{X})\neq 0\;and\;f(w_{k,t}^{Y})\neq 0\\ 1 & if\;f(w_{k,t}^{X})=0\;and\;f(w_{k,t}^{Y})\neq 0\\ 1 & if\;f(w_{k,t}^{X})\neq 0\;and\;f(w_{k,t}^{Y})=0\\ 0 & if\;f(w_{k,t}^{X})=0\;and\;f(w_{k,t}^{Y})=0 \end{array} \end{array}.$$

In the definition of function *score*, the frequencies of all the *k*-words in protein sequence are considered. Indeed, the measure *gsm.k *is the edit score between *k*-word frequencies of the two protein sequences *X *and *Y*. If a *k*-word *w *appears in the two sequences, the edit score is $f(w_{k,w}^{X})\cdot \log_{2}\left(\frac{2\cdot f(w_{k,w}^{X})}{f(w_{k,w}^{X})+f(w_{k,w}^{Y})}\right)$. If a *k*-word *w *appears in protein sequence *X *not *Y*, it seems that the *k*-word *w *is deleted from the protein sequence *Y*, we choose the maximum value of function $f(w_{k,w}^{X})\cdot \log_{2}\left(\frac{2\cdot f(w_{k,w}^{X})}{f(w_{k,w}^{X})+f(w_{k,w}^{Y})}\right)$ as the gap penalty according to followed proposition.

**Proposition**. If $F_{k}^{A}=\left(f(w_{k,1}^{A}),f(w_{k,1}^{A}),\cdots ,f(w_{k,n}^{A})\right)$*and *$F_{k}^{B}=\left(f(w_{k,1}^{B}),f(w_{k,1}^{B}),\cdots ,f(w_{k,n}^{B})\right)$ are two *k*-word frequency vectors of length *n*,

(20) $$f(w_{k,t}^{X})\cdot \log_{2}\left(\frac{2\cdot f(w_{k,t}^{X})}{f(w_{k,t}^{X})+f(w_{k,t}^{Y})}\right)\leq 1.$$

**Proof: **To find its maximum, we rewrite

$$\begin{array}{l} f(w_{k,t}^{X})\cdot \log_{2}\left(\frac{2\cdot f(w_{k,t}^{X})}{f(w_{k,t}^{X})+f(w_{k,t}^{Y})}\right)\\ =f(w_{k,t}^{X})\cdot \log_{2}2+f(w_{k,t}^{X})\cdot \log_{2}\left(\frac{f(w_{k,t}^{X})}{f(w_{k,t}^{X})+f(w_{k,t}^{Y})}\right) \end{array}.$$

Since $\frac{f(w_{k,t}^{X})}{f(w_{k,t}^{X})+f(w_{k,t}^{Y})}\leq 1$, we can get

$$\log_{2}\left(\frac{f(w_{k,t}^{X})}{f(w_{k,t}^{X})+f(w_{k,t}^{Y})}\right)\leq 0.$$

Thus

$$f(w_{k,t}^{X})\cdot \log_{2}\left(\frac{2\cdot f(w_{k,t}^{X})}{f(w_{k,t}^{X})+f(w_{k,t}^{Y})}\right)\leq 1.$$

Similarly, the symmetric form of *gsm.k*, denoted as *Gdis.k*, between two sequences *X *and *Y *is defined by

(21) $$\begin{array}{l} Gdis.k(X,Y)\\ =\{\begin{array}{cc} 0 & if\;F_{k}^{X}=F_{k}^{Y}\\ (gsm(X,Y)+gsm(Y,X))/n+2 & else \end{array} \end{array}.$$

A distance metric, D(·,·), should satisfy the following conditions:

1. *D*(*S*, *Q*) ≥ 0, where the equality is satisfied iff *S *= *Q *(identity).

2. *D*(*S*, *Q*) = *D*(*Q*, *S*)(symmetry).

3. *D*(*S*, *Q*) ≤ *D*(*S*, *T*) + *D*(*T*, *Q*)(triangle inequality).

In the appendix, we prove that the statistical measure, *Gdis.k*, defined above satisfies the three conditions and is, therefore, a valid distance metric.

### Evaluation methods

Similarity/dissimilarity measures are compared by considering how well they classify protein sequences, as well as by computing receiver operator characteristic (ROC) curves. ROC goes back to signal detection and classification problems and is now widely used [47]. This approach is employed in binary classification of continuous data, usually categorized as positive (1) or negative (0) cases. The classification accuracy can be measured by plotting, for different threshold values, the number of true positives (TP), also named sensitivity or coverage versus false positives (FP), or (1-specificity), encountered for each threshold, properly normalized [Eq. 22].

(22) $$\begin{array}{r} sensitivity=\frac{TruePositives}{Positives}=\frac{TP}{TP+FN},\\ specificity=\frac{TrueNegatives}{Negatives}=\frac{TN}{TN+FP},\\ 1-specificity=\frac{FP}{TN+FP}. \end{array}$$

A ROC curve is simply the plot of sensitivity versus (1-specificity) for different threshold values. The area under a ROC curve (AUC) is a widely employed parameter to quantify the quality of a classificator because it is a threshold independent performance measure and is closely related to the Wilcoxon signed-rank test [48]. For a perfect classifier, the AUC is 1 and for a random classifier the AUC is 0.5

## Availability

Software name: SMPS-SS

Software home page: 

Operating system(s): windows

Programming languages: perl

License: web server freely available without registration

Restrictions to use by non-academics: on request

## Abbreviations

AUC: Area Under the Curve; b..: BLOSUM..; CATH: Hierarchical Classification of Protein Domain Structures; CD-HIT: Cluster Database at High Identity with Tolerance; CK: Chew-Kedem Data; cos.*k*: Cosine of the Angle Based on *k*-word Frequencies of Protein Sequence; cos,*k*.matrix: Cosine of the Angle Based on *k*-word Frequencies of Protein 'Sequence Space' Constructed According to Score Matrix; DAUC: Different Area under the Curve; ed.*k*: Euclidian Distance Based on *k*-word Frequencies of Protein Sequence; ed.*k*.matrix: Euclidian Distance Based on *k*-word Frequencies of Protein 'Sequence Space' Constructed According to Score Matrix; FP: False Positives; Gdis.*k*: Gapped Distance Measure Based on *k*-word Frequencies; gre.*k*: Generalized Relative Entropy Based on *k*-word Frequencies of Protein Sequence; gre.*k*.matrix: Generalized Relative Entropy Based on *k*-word Frequencies of Protein 'Sequence Space' Constructed According to Score Matrix; gsm.*k*: Gapped Similarity Measure Based on *k*-word Frequencies of Protein Sequence; gsm.*k*.matrix: Gapped Similarity Measure Based on *k*-word Frequencies of Protein 'Sequence Space' Constructed According to Score Matrix; kld: Kullback-Leibler Discrepancy; MAUC: Maximal Area under the Curve; MEGA: Molecular Evolutionary Genetics Analysis; NW: Needleman-Wunsch Measure; NW-inear.matrix: Needleman-Wunsch Measure Using Score Matrix and Linear Gap Penalty; NW-affine.matrix: Needleman-Wunsch Measure Using Score Matrix and Affine Gap Penalty; p..: PAM..; pfam: Protein Family; PIR: Protein Information Resource; ROC: Receiver Operating Curve; RS: Rost and Sander Data; s1.k: S1 Measure Based on *k*-word Frequencies of Protein Sequence; s1.matrix: S1 Measure Based on *k*-word Frequencies of Protein 'Sequence Space' Constructed According to Score Matrix; s2.k: S2 Measure Based on *k*-word Frequencies of Protein Sequence; s2.k.matrix: S2 Measure Based on *k*-word Frequencies of Protein 'Sequence Space' Constructed According to Score Matrix; SCOP: Structural Classification of Proteins; se.k: Standardized Euclidean Distance Based on *k*-word Frequencies of Protein Sequence; se.k.matrix: Standardized Euclidean Distance Based on *k*-word Frequencies of Protein 'Sequence Space' Constructed According to Score Matrix; SM: Smith-Waterman Measure; SM-linear.matrix: Smith-Waterman Measure Using Score Matrix and Linear Gap Penalty; SM-affine.matrix: Smith-Waterman Measure Using Score Matrix and Affine Gap Penalty; SMC: Structural Maintenance of Chromosomes; SP: Sierk-Pearson Data; SPs: 'Sequence Space' of Sequence *s*; SS.k: Similarity Score Based on *k*-word Frequencies; Swiss-Prot: Swiss-Prot Database; TP: True Positives; W.k.matrix: W-metric Based on *k*-word Frequencies and Score Matrix;

## Authors' contributions

QD conceived the method and prepared the manuscript. QD implemented the software and performed the ROC analysis. QD and TMW contributed to the discussion and have approved the final manuscript.

## Appendix

### The proof of valid distance metric

**Lemma 1**. For a real convex function *f *in its domain [a, b], ∀ *x*_*i *_∈ [*a*, *b*], *λ*_*i *_> 0 (i = 1, 2, ⋯, *n*), $\sum_{i=1}^{n}\lambda_{i}=1$, Jensen's inequality can be stated as:

(23) $$f(\sum_{i=1}^{n}\lambda_{i}x_{i})\leq \sum_{i=1}^{n}\lambda_{i}f(x_{i}).$$

**Proof: **Let $x_{0}=\sum_{i=1}^{n}\lambda_{i}x_{i}$, *x*_0 _∈ [*s*, *b*]. We expand f(x) around *x*_0_, and by Taylor's theorem, we have that

$$\begin{array}{cc} \begin{array}{c} f(x)=f(x_{0})+f^{\prime }(x_{0})(x-x_{0})\\ +\frac{f^{\prime \prime }(\xi )}{2!}{\left(x-x_{0}\right)}^{2} \end{array}, & \xi \in [a,b]. \end{array}$$

Since f(x) is a real convex function f in its domain [a, b], *f*" (*ξ*) > 0. Thus we have

*f*(*x*) ≥ *f*(*x*_0_) + *f*'(*x*_0_)(*x *- *x*_0_).

For all *x*_*i *_∈ [*a*, *b*], we can obtain that

$$\begin{array}{c} f(x_{1})\geq f(x_{0})+f^{\prime \prime }(x_{0})(x_{1}-x_{0}),\\ f(x_{2})\geq f(x_{0})+f^{\prime \prime }(x_{0})(x_{2}-x_{0}),\\ \vdots \\ f(x_{n})\geq f(x_{0})+f^{\prime \prime }(x_{0})(x_{n}-x_{0}). \end{array}$$

Multiplying the above inequalities with *λ*_*i*_, we have

$$\begin{array}{c} \lambda_{1}\cdot f(x_{1})\geq \lambda_{1}\cdot f(x_{0})+\lambda_{1}\cdot f^{\prime \prime }(x_{0})(x_{1}-x_{0}),\\ \lambda_{2}\cdot f(x_{2})\geq \lambda_{2}\cdot f(x_{0})+\lambda_{2}\cdot f^{\prime \prime }(x_{0})(x_{2}-x_{0}),\\ \vdots \\ \lambda_{n}\cdot f(x_{n})\geq \lambda_{n}\cdot f(x_{0})+\lambda_{n}\cdot f^{\prime \prime }(x_{0})(x_{n}-x_{0}). \end{array}$$

Summing the above inequalities,

$$\begin{array}{c} \sum_{i=1}^{n}\lambda_{i}f(x_{i})\geq f(x_{0})\sum_{i=1}^{n}\lambda_{i}+\sum_{i=1}^{n}\lambda_{i}f^{\prime }(x_{0})(x_{i}-x_{0})\\ =f(x_{0}). \end{array}$$

Thus, we obtain that

$$f(\sum_{i=1}^{n}\lambda_{i}x_{i})\leq \sum_{i=1}^{n}\lambda_{i}f(x_{i}).$$

**Proposition 1**. ∀ *x*, *y *> 0,

(24) $$(x+y)\ln\left(\frac{x+Y}{2}\right)\leq x\ln x+y\ln y.$$

**Proof: **Let *f(x) *= *xlnx*, *x *> 0, we calculate *f*'(*x*) and *f*"(*x*),

$$\begin{array}{cc} f^{\prime }(x)=\ln x=1, & f^{\prime \prime }(x)=\frac{1}{x}. \end{array}$$

Thus *f*(*x*) is a real convex function.

According to Lemma 1, we have

$$\frac{x+y}{2}\ln\left(\frac{x+Y}{2}\right)\leq \frac{1}{2}(x\ln x+y\ln y).$$

Then

$$(x+y)\ln\left(\frac{x+Y}{2}\right)\leq (x\ln x+y\ln y).$$

If $F_{k}^{A}=\left(f(w_{k,1}^{A}),f(w_{k,1}^{A}),\cdots ,f(w_{k,n}^{A})\right)$*and *$F_{k}^{B}=\left(f(w_{k,1}^{B}),f(w_{k,1}^{B}),\cdots ,f(w_{k,n}^{B})\right)$ are two *k*-word frequency vectors of protein sequences *X *and *Y*, respectively, we define similarity score, denoted by *ss.k*, as follows:

(25) $$\begin{array}{c} ss.k(X,Y)=\sum_{t=1}^{n}score(f(w_{k,t}^{X}),f(w_{k,t}^{Y}))\\ +\sum_{t=1}^{n}score(f(w_{k,t}^{Y}),f(w_{k,t}^{X})) \end{array},$$

where

$$\begin{array}{l} score(f(w_{k,t}^{I}),f(w_{k,t}^{J}))\\ =\{\begin{array}{cc} f(w_{k,t}^{I})\cdot \log_{2}\left(\frac{2\cdot f(w_{k,t}^{I})}{f(w_{k,t}^{I})+f(w_{k,t}^{J})}\right) & if\;f(w_{k,t}^{I})\neq 0\;and\;f(w_{k,t}^{J})\neq 0\\ 1 & if\;f(w_{k,t}^{I})=0\;and\;f(w_{k,t}^{J})\neq 0\\ 1 & if\;f(w_{k,t}^{I})\neq 0\;and\;f(w_{k,t}^{J})=0\\ 0 & if\;f(w_{k,t}^{I})=0\;and\;f(w_{k,t}^{J})=0 \end{array} \end{array}$$

**Proposition 2**.

(26) $$0\leq score(f(w_{k,t}^{I}),f(w_{k,t}^{J}))+f(w_{k,t}^{I}),f(w_{k,t}^{J})\leq 2.$$

**Proof**: Firstly, we need to show that

(27) $$score(f(w_{k,t}^{I}),f(w_{k,t}^{J}))+f(w_{k,t}^{J}),f(w_{k,t}^{I})\geq 0.$$

*Case 1*: $f(w_{k,t}^{I})$ = $f(w_{k,t}^{J})$ = 0, it satisfies the above inequality.

*Case 2*: The entry of $f(w_{k,t}^{I})$ or $f(w_{k,t}^{J})$ is equal to zero. Without loss of generality, assume $f(w_{k,t}^{I})$ = 0 and $f(w_{k,t}^{J})$ ≠ 0, we can easily get that

$$score(f(w_{k,t}^{I}),f(w_{k,t}^{J}))+f(w_{k,t}^{J}),f(w_{k,t}^{I})=2>0.$$

*Case 3*: $f(w_{k,t}^{I})$ ≠ 0 and $f(w_{k,t}^{J})$ ≠ 0. Using the Proposition 1, we can easily obtain the inequality (24).

To find its maximum, we use the Proposition in Method section to get that

$$score(f(w_{k,t}^{I}),f(w_{k,t}^{J}))+f(w_{k,t}^{I}),f(w_{k,t}^{J})\leq 2.$$

**Theorem 1**. The statistical measure *Gdis.k(X,Y) *is a distance metric.

**Proof**: Again, by definition *ss.k*(*X*, *Y*) and Proposition 2, we can obtain that it satisfies two important mathematical properties: (1) positivity: *Gdis.k*(*X*, *Y*) ≥ 0 and *Gdis.k*(*X*, *Y*) = 0 ⇔ $F_{k}^{X}$ = $F_{k}^{Y}$; (2) symmetry: *Gdis.k*(*X*, *Y*) = *Gdis.k*(*Y*, *X*). We now need to show that *Gdis.k*(*X*, *Y*) ≥ 0 satisfies the triangle inequality:

*Gdis.k*(*X*, *Y*) ≤ *Gdis.k*(*X*, *Z*) + *Gdis.k*(*Z*, *Y*).

Case 1: $F_{k}^{X}$ = $F_{k}^{Y}$ = $F_{k}^{Z}$, it satisfies the triangle inequality.

Case 2: Among three *k*-word frequency vectors, two vectors are equal. Without loss of generality, assume $F_{k}^{X}$ ≠ $F_{k}^{Y}$ and $F_{k}^{X}$ = $F_{k}^{Z}$, we can easily obtain that

*Gdis.k*(*X*, *Y*) ≤ *Gdis.k*(*X*, *Z*) + *Gdis.k*(*Z*, *Y*).

Case 3: $F_{k}^{X}$ ≠ $F_{k}^{Y}$ ≠ $F_{k}^{Z}$. From the definition of *ss.k *and Proposition 2, we have

$$\begin{array}{l} Gdis.k(X,Z)+Gdis.k(Z,Y)\\ =ss.k(X,Z)/n+ss.k(Z,Y)/n+4\geq 4 \end{array}.$$

Since

*Gdis.k*(*X*, *Y*) = *ss.k*(*X*, *Y*)/*n *+ 2 ≤ 4.

Thus

*Gdis.k*(*X*, *Y*) ≤ *Gdis.k*(*X*, *Z*) + *Gdis.k*(*Z*, *Y*).

## Supplementary Material

Additional file 1 **The Chew-Kedem data set**. The protein sequences used in Chew-Kedem data with the accession numbers of PDB. Click here for file

Additional file 2 **The Rost-Sander dataset**. The protein sequences used in Rost-Sander data with the accession numbers of PDB. Click here for file

Additional file 3 **The Sierk-Pearson data**. The protein sequences used in Sierk-Pearson data with the accession numbers of PDB. Click here for file

Additional file 4 **The protein data used in phylogenetic analysis**. The protein sequences used in phylogenetic analysis with abbreviated names, full names and Accession numbers. Click here for file

## References

[B1] Bateman A, Birney E, Cerruti L, Durbin R, Etwiller L, Eddy SR, Griffiths JS, Howe KL, Marshall M, Sonnhammer ELL (2002). The Pfam Protein FamiliesDatabase. Nucleic Acids Res.

[B2] Andreeva A, Howorth D, Brenner SE, Hubbard TJP, Chothia C, Murzin AG (2004). SCOP database in refinements integrate structure and sequence family data. Nucleic Acid Res.

[B3] Bairoch A, Apweiler R (2000). The SWISS-PROT protein sequence database and its supplement TrEMBL in 2000. Nucleic Acids Res.

[B4] Wu CH, Huang H, Arminski L, Castro-Alvear J, Chen Y, Hu ZZ, Ledley RS, Lewis KG, Mewes HW, Orcutt BC, Suzek BE, Tsugita A, Vinayaka CR, Yeh LSL, Zhang J, Barker WC (2002). The Protein Information Resource, an integrated public resource of functional annotation of proteins. Nucleic Acids Res.

[B5] Altschul SF, Gish W, Miller W, Myers EW, Lipman DJ (1990). Basic local alignment search tool. J Mol Biol.

[B6] Altschul SF, Madden TL, Schaffer AA, Zhang J, Zhang Z, Miller W, Lipman DJ (1997). Gapped BLAST and PSI-BLAST: a new generation of protein database search programs. Nucleic Acids Res.

[B7] Pham TD (2007). Spectral distortion measures for biological sequence comparisons and database searching. Pattern Recog.

[B8] Felsenstein J (1981). Evolutionary trees from DNA sequences, a maximum likelihood approach. J Mol Evol.

[B9] Felsenstein J (1996). Inferring phylogenies from protein sequences by parsimony, distance and likelihood methods. Meth Enzymol.

[B10] Huelsenbeck JP, Ronquist F (2001). MRBAYES: Bayesian inference of phylogenetic trees. Bioinformatics.

[B11] Kumar S, Tamura K, Nei M (2004). MEGA3: integrated software for molecular evolutionary genetics analysis and sequence alignment. Brief Bioinform.

[B12] Ronquist F, Huelsenbeck JP (2003). MrBayes 3: Bayesian phylogenetic inference under mixed models. Bioinformatics.

[B13] Komatsu K, Zhu S, Fushimi H, Qui TK, Cai S, Kadota S (2001). Phylogenetic analysis based on 18S rRNA gene and matK gene sequences of Panax vietnamensis and five related species. Planta Med.

[B14] Vinga S, Gouveia-Oliveira R, Almeida JS (2004). Comparative evaluation of word composition distances for the recognition of SCOP relationships. Bioinformatics.

[B15] Ferragina P, Giancarlo R, Greco V, Manzini G, Valiente G (2007). Compression-based classification of biological sequences and structures via the Universal Similarity Metric: experimental assessment. BMC Bioinformatics.

[B16] Vinga S, Almeida J (2003). Alignment-free sequence comparison – a review. Bioinformatics.

[B17] Pham TD, Zuegg J (2004). A probabilistic measure for alignment-free sequence comparison. Bioinformatics.

[B18] Blaisdell BE (1986). A measure of the similarity of sets of sequences not requiring sequence alignmen. Proc Natl Acad Sci USA.

[B19] Wu TJ, Burke JP, Davison DB (1997). A measure of DNA sequence dissimilarity based on Mahalanobis distance between frequencies of words. Biometrics.

[B20] Wu TJ, Hsieh YC, Li LA (2001). Statistical measures of DNA dissimilarity under Markov chain models of base composition. Biometrics.

[B21] Stuart GW, Moffett K, Baker S (2002). Integrated gene and species phylogenies from unaligned whole genome protein sequences. Bioinformatics.

[B22] Fichant G, Gautier C (1987). Statistical method for predicting protein coding regions in nucleic acid sequences. Comput Appl Biosci.

[B23] Wu KP, Lin HN, Sung TY, Hsu WL (2003). A New Similarity Measure among Protein Sequences. Proceedings of IEEE CSB2003 Computer Society Bioinformatics Conference.

[B24] Didier G, Laprevotte I, Pupin M, Hénaut A (2006). Local decoding of sequences and alignment-free comparison. J Comput Biol.

[B25] Kelil A, Wang S, Brzezinski R, Fleury A (2007). CLUSS: Clustering of Protein Sequences Based on a New Similarity Measure. BMC Bioinformatics.

[B26] Hochreiter S, Heusel M, Obermayer K (2007). Fast model-based protein homology detection without alignment. Bioinformatics.

[B27] Chew LP, Kedem K (2003). Finding the Consensus Shape for a Protein Family. Algorithmica.

[B28] Sierk M, Person W (2004). Sensitivity and Selectivity in Protein Structure Comparison. Protein Sci.

[B29] Thiruv B, Quon G, Saldanha SA, Steipe B (2005). Nh3D: A Reference Dataset of Non-Homologous Protein Structures. BMC Struct Biol.

[B30] Word JM, Lovell SC, LaBean TH, Taylor HC, Zalis ME, Presley BK, Richardson JS, Richardson DC (1999). Visualizing and Quantifying Molecular Goodness-of-Fit: Small-Probe Contact Dots with Explicit Hydrogen Atoms. J Mol Biol.

[B31] Krasnogor N, Pelta DA (2004). Measuring the Similarity of Protein Structures by Means of the Universal Similarity Metric. Bioinformatics.

[B32] Rost B, Sander C (1993). Prediction of protein secondary structure at better than 70% accuracy. J Mol Biol.

[B33] Barthel D, Hirst JD, Blażewicz J, Burke EK, Krasnogor N (2007). ProCKSI: A Decision Support System for Protein (Structure) Comparison, Knowledge, Similarity and Information. BMC Bioinformatics.

[B34] SCOP: Structural Classification of Proteins. http://scop.mrclmb. cam.ac.uk/scop.

[B35] Pearl F (2005). The CATH Domain Structure Database and Related Resources Gene3D and DHS Provide Comprehensive Domain Family Information for Genome Analysis. Nucleic Acids Res.

[B36] Li W, Godzik A, Cd-hit (2006). A fast program for clustering and comparing large sets of protein or nucleotide sequences. Bioinformatics.

[B37] Felsenstein J (1989). PHYLIP-Phylogeny inference package (version 3.2). Cladistics.

[B38] Saitoh N, Goldberg I, Earnshaw WC (1995). The SMC proteins and the coming of age of the chromosome scaffold hypothesis. BioEssays.

[B39] Lowe J, Cordell SC, Ent F Van Den (2001). Crystal structure of the SMC head domain: an ABC ATPase with 900 residues antiparallel coiled-coil inserted. J Mol Biol.

[B40] Hirano M, Hirano T (2002). Hinge-mediated dimerization of SMC protein is essential for its dynamic interaction with DNA. EMBO J.

[B41] Cobbe N, Heck MM (2000). SMCs in the world of chromosome biology- from prokaryotes to higher eukaryotes. J Struct Biol.

[B42] Soppa J (2001). Prokaryotic structural maintenance of chromosomes (SMC) proteins: distribution, phylogeny, and comparison with MukBs and additional prokaryotic and eukaryotic coiled-coil proteins. Gene.

[B43] Taylor EM, Moghraby JS, Lees JH, Smit B, Moens PB, Lehmann AR (2001). Characterization of a novel human SMC heterodimer homologous to the Schizosaccharomyces pombe Rad18/Spr18 complex. Mol Biol Cell.

[B44] Fujioka Y, Kimata Y, Nomaguchi K, Watanabe K, Kohno K (2002). Identification of a novel non-SMC component of the SMC5/SMC6 complex involved in DNA repair. J Biol Chem.

[B45] Reinert G, Schbath S, Waterman MS (2000). Probabilistic and statistical properties of words: an overview. J Comput Biol.

[B46] Kroupa T Measure of divergence of possibility measures. Proceedings of the 6th Workshop on Uncertainty Processing (WUPES'2003), Hejnice, Czech Republic.

[B47] Egan JP (1975). Signal Detection Theory and ROC-Analysis.

[B48] Bradley AP (1997). The use of the area under the ROC curve in the evaluation of machine learning algorithms. Pattern Recog.

