# Supplementary material for: Comparison study on k-word statistical measures for protein: From sequence to 'sequence space'
Source: BMC Bioinformatics. 2008 Sep 23;9:394. doi: 10.1186/1471-2105-9-394 (PMC2571980; doi:10.1186/1471-2105-9-394)
Supplement: Additional file 1 — The Chew-Kedem data set. The protein sequences used in Chew-Kedem data with the accession numbers of PDB. [file 1471-2105-9-394-S1.pdf]

The Chew-Kedem data set consists of the following proteins:

**1. mainly alpha**

1ash00, 1babA0, 1babB0, 1cnpA0, 1eca00, 1flp00, 1hlb00, 1hlm00, 1ithA0, 1jhgA0, 1lh200, 1mba00, 1myt00, 2hbg00, 2lhb00, 2vhb00, 2vhbA0, 3sdhA0, 5mbn00.

**2. mainly beta**

1cd800, 1cdb00, 1ci5A0, 1hnf01, 1neu00, 1qa9A0, 1qfoA0.

**3. alpha-beta:**

1aa900, 1chrA1, 1ct9A1, 1gnp00, 1qraA0, 2mnr01, 4enl01, 5p2100, 6q21A0, 6xia00.
