# Supplementary material for: Comparison study on k-word statistical measures for protein: From sequence to 'sequence space'
Source: BMC Bioinformatics. 2008 Sep 23;9:394. doi: 10.1186/1471-2105-9-394 (PMC2571980; doi:10.1186/1471-2105-9-394)
Supplement: Additional file 2 — The Rost-Sander dataset. The protein sequences used in Rost-Sander data with the accession numbers of PDB. [file 1471-2105-9-394-S2.pdf]

The Rost and Sander dataset (RS121) was designed for the secondary structure prediction of proteins with a pair-wise sequence similarity of less than 25%. We adopt this manually curated database as our gold standard containing expert knowledge for class level. This dataset is trimmed to exclude sequences belonging to classes with <5 elements, thus obtaining the protein dataset of size 121 named RS.

1. All alpha proteins

1cc5A,1ecaA, 1gdj, 1lmb3, 2ccy, 2cyp, 2lhb, 2or1l, 2tmvp, 2utga, 2wrpr, 3cln , 3gapa, 3icb , 4bp2 , 4cpv , 4sdha, 5cytr, 6cpp , 6cts , 256ba.

2. All beta proteins

1acxA, 1azuA, 1bbpA, 1bmv1, 1bmv2, 1fc2C, 1fdlH, 1fndA, 1fndA, 1mcp, 1paz, 1pyp, 1r092, 1rbp , 1tgsi, 1tnfa, 2alp, 2cab, 2gcr, 2gn5, 2hmza, 2i1b, 2ltna, 2ltnb, 2mev4, 2paba, 2pcy, 2rspa, 2sns, 2sodb, 2stv, 2tgpi, 3ait, 3cd4, 3hmga, 3hmgb, 4cms, 4rhv1, 4rhv3, 4rhv4, 4sgbi, 5er2e, 5hvpa, 8adh.

3. Alpha and beta proteins (a+b)

1durA, 1fkfA, 1fxiA, 1il8a, 1l58, 1ubq, 2fxb, 2glsa, 2tsca, 3b5c, 3rnt, 5lyz, 6tmne, 7rsa, 9pap.

4. Alpha and beta proteins (a/b)

1cseI, 1etuA, 1gd1o, 1gp1a, 1lap, 1rhd, 1s01, 1wsya, 1wsyb, 2aat, 2ak3a, 2fox, 2gbp, 2phh, 3cla, 3pgm, 3tima, 4cpai, 4gr1, 4pfk, 4ts1a, 4xiaa, 5ldh, 6acn, 6cpa, 6dfr, 7icd, 8abp.

5. Small proteins

1bdsA, 1cbhA, 1cdtA, 1crnA, 1hip, 1mrt, 1ovoa, 1sh1, 2mhu, 4rxn, 6hir, 9insb,9wgaa.
