# Supplementary material for: Comparison study on k-word statistical measures for protein: From sequence to 'sequence space'
Source: BMC Bioinformatics. 2008 Sep 23;9:394. doi: 10.1186/1471-2105-9-394 (PMC2571980; doi:10.1186/1471-2105-9-394)
Supplement: Additional file 3 — The Sierk-Pearson data. The protein sequences used in Sierk-Pearson data with the accession numbers of PDB. [file 1471-2105-9-394-S3.pdf]

The Sierk-Pearson protein data set is as follows:

1. mainly alpha

1ad600, 1ao6A5, 1bbhA0, 1cnsA1, 1d2zD0, 1dat00, 1e12A0, 1eqzE0, 1gwxA0, 1hgu00, 1hlm00, 1jnk02, 1mmoD0, 1nubA0, 1quuA1, 1repC1, 1sw6A0, 1trrA0, 2hpdA0, 2mtaC0.

2. mainly beta

1a8d02, 1a8h02, 1aozA3, 1b8mB0, 1bf203, 1bjqB0, 1bqyA2, 1btkB0, 1c1zA5, 1cl7H0, 1d3sA0,1danU0, 1dsyA0, 1dxmA0, 1et6A2, 1extB1, 1nfiC1, 1nukA0, 1otcA1, 1qdmA2, 1qe6D0, 1qfkL2, 1que01, 1rmg00, 1tmo04, 2tbvC0.

3. alpha-beta

1a1mA1, 1a2vA2, 1akn00, 1aqzB0, 1asyA2, 1atiA2, 1auq00, 1ax4A1, 1b0pA6, 1b2rA2, 1bcg00, 1bcmA1, 1bf5A4, 1bkcE0, 1bp7A0, 1c4kA2, 1cd2A0, 1cdg01, 1d0nA4, 1d4oA0, 1d7oA0, 1doi00, 1dy0A0, 1e2kB0, 1eccA1, 1fbnA0, 1gsoA3, 1mpyA2, 1obr00, 1p3801, 1pty00, 1qb7A0, 1qmvA0, 1urnA0, 1zfjA0, 2acy00, 2drpA1, 2nmtA2, 2reb01, 4mdhA2.
