# Supplementary material for: Comparison study on k-word statistical measures for protein: From sequence to 'sequence space'
Source: BMC Bioinformatics. 2008 Sep 23;9:394. doi: 10.1186/1471-2105-9-394 (PMC2571980; doi:10.1186/1471-2105-9-394)
Supplement: Additional file 4 — The protein data used in phylogenetic analysis. The protein sequences used in phylogenetic analysis with abbreviated names, full names and Accession numbers. [file 1471-2105-9-394-S4.pdf]

Descriptions: The protein sequences used in phylogenetic analysis with abbreviated names, full names and Accession numbers.

The abbreviated name	The full name	Accession number
**MukB**		
E.colMukB	Escherichia coli MukB	AAC74010
K.pneMukB	Klebsiella pneumoniae MukB	CAD10421
P.mulMukB	Pasteurella multocida MukB	NP_245546
S.typMukB	Salmonella typhimurium MukB	CAD10422
Y.pesMukB	Yersinia pestis MukB	NP_404998
**Rad50**		
A.thRad50	Arabidopsis thaliana Rad50	AAF36810
C.elRad50	Caenorhabditis elegansRad50	CAB01581
D.meRad50	Drosophila melanogaster Rad50	AAF46847
H.saRad50	Homo sapiens Rad50	CAA99729
S.ceRad50	Saccharomyces cerevisiae Rad50	BWBYDL
**SMC**		
A.fulSMC	Archaeoglobus fulgidus SMC	AAB89690
B.perSMC	Bordetella pertussis SMC	CAD66592
B.cepSMC	Burkholderia cepacia SMC	CAD10419
C.tepSMC	Chlorobium tepidum SMC	AAM72931
G.sulSMC	Geobacter sulfurreducens SMC	CAD66600
M.capSMC	Methylococcus capsulatus SMC	CAD66601
N.menSMC	Neisseria meningitidis SMC	AAF40974
N.eurSMC	Nitrosomonas europaea SMC	BN000118
P.aerSMC	Pseudomonas aeruginosa SMC	D83454
P.furSMC	Pyrococcus furiosus SMC	CAD66602
S.oneSMC	Shewanella oneidensis SMC	NP_718470
S.sp.SMC	Synechococcus sp. PCC 7002 SMC	CAD66604
T.volSMC	Thermoplasma volcanium SMC	BAB59963
T.eloSMC	Thermosynechococcus elongatus SMC	BAC09477
**SMC1**		
A.gamSMC1	Anopheles gambiae SMC1	CAD59403

A.thaSMC1	Arabidopsis thaliana SMC1	CAB77587
B.tauSMC1	Bos taurus SMC1	AAD13141
C.eleSMC1	Caenorhabditis elegans SMC1	NP_491486
D.melSMC1	Drosophila melanogaster SMC1	CAB76376
G.galSMC1	Gallus gallus SMC1	CAD58850
H.sa1SMC1	Homo sapiens SMC1	Q14683
H.sa2SMC1	Homo sapiens SMC1beta	CAD43404
L.majSMC1	Leishmania major SMC1	AAK64500
**SMC2**		
A.gamSMC2	Anopheles gambiae SMC2	CAD59404
A.thaSMC2	Arabidopsis thaliana SMC2b	CAB61972
C.eleSMC2	Caenorhabditis elegans SMC2	AAC47834
D.melSMC2	Drosophila melanogaster SMC2	AAD52673
M.musSMC2	Mus musculus SMC2	CAD59182
O.satSMC2	Oryza sativa SMC2	CAD59410
S.cerSMC2	Saccharomyces cerevisiae SMC2	P38989
T.rubSMC2	Takifugu rubripes SMC3	CAD58849
**SMC3**		
A.gamSMC3	Anopheles gambiae SMC3	CAD59405
A.thaSMC3	Arabidopsis thaliana SMC3	CAD43403
B.tauSMC3	Bos taurus SMC3	BN000098
C.eleSMC3	Caenorhabditis elegans SMC3	CAB57898
D.melSMC3	Drosophila melanogaster SMC3	AAC47078
G.galSMC3	Gallus gallus SMC3	CAD58708
H.sapSMC3	Homo sapiens SMC3	NP_005436
M.musSMC3	Mus musculus SMC3	AAD27754
O.satSMC3	Oryza sativa SMC3	CAD59411
R.norSMC3	Rattus norvegicus SMC3	AAB96342
**SMC4**		
A.gamSMC4	Anopheles gambiae SMC4	CAD59406
A.thaSMC4	Arabidopsis thaliana SMC4	BAB10693
C.eleSMC4	Caenorhabditis elegans SMC4	CAA86336
D.melSMC4	Drosophila melanogaster SMC4	AAF01416

G.galSMC4	Gallus gallus SMC4	CAD58707
L.majSMC4	Leishmania major SMC4	CAB95273
M.arvSMC4	Microtus arvalis SMC4	CAC09583
M.musSMC4	Mus musculus SMC4	CAD59183
O.satSMC4	Oryza sativa SMC4	CAD32690
**SMC5**		
A.gamSMC5	Anopheles gambiae SMC5	CAD59407
A.fumSMC5	Aspergillus fumigatus SMC5	AAL82734
C.eleSMC5	Caenorhabditis elegans SMC5	NP_494935
D.melSMC5	Drosophila melanogaster SMC5	CAD29584
H.sapSMC5	Homo sapiens SMC5	CAC39247
M.musSMC5	Mus musculus SMC5	CAD59184
O.satSMC5	Oryza sativa SMC5	CAD59412
S.cerSMC5	Saccharomyces cerevisiae SMC5	NP_014608
T.rubSMC5	Takifugu rubripes SMC5	CAD65850
SMC6		
A.gamSMC6	Anopheles gambiae SMC6	CAD59408
A.thaSMC6	Arabidopsis thaliana SMC6b	BAB11444
C.el1SMC6	Caenorhabditis elegans SMC6a	CAB01681
C.el2SMC6	Caenorhabditis elegans SMC6b	CAB16920
D.melSMC6	Drosophila melanogaster SMC6	NP_651228
H.sapSMC6	Homo sapiens SMC6	CAC39248
M.musSMC6	Mus musculus SMC6	CAC39250
O.satSMC6	Oryza sativa SMC6	CAD59413
T.rubSMC6	Takifugu rubripes SMC6	CAD65851
